# Strengthening preparedness and response to emerging henipavirus diversity

**DOI:** 10.3389/fcimb.2026.1761347

**Published:** 2026-02-25

**Authors:** Kok Keng Tee, Xueshan Xia

**Affiliations:** Yunnan Province Key Laboratory of Public Health and Biosafety, School of Public Health, Kunming Medical University, Kunming, Yunnan, China

**Keywords:** emerging infectious diseases, henipavirus, nipah virus, one health, public health surveillance, zoonoses

## Abstract

Henipaviruses, including the highly pathogenic Nipah virus and Hendra virus, represent a major zoonotic threat with high mortality rates and potential for human-to-human transmission. Recent discoveries of novel henipaviruses in China, Europe and other regions highlight the urgent need for enhanced surveillance in both wildlife reservoirs such as bats, shrews, rodents, and human populations, particularly in high-risk areas. Despite advancements in metagenomic sequencing, gaps in integrated surveillance, fragmented One Health implementation, and insufficient diagnostic infrastructure in large parts of the world hinder global preparedness. This paper identifies key challenges in henipavirus detection and control and proposes an operational roadmap for surveillance, diagnostics, and cross-sectoral collaboration. With the known animal hosts of henipaviruses and related henipa-like orthoparamyxoviruses now documented across more than 130 countries and territories, strengthening these capabilities is critical to preventing future epidemics and addressing the evolving threat of emerging henipavirus diversity.

## Introduction

Henipaviruses belong to the *Paramyxoviridae* family (*Orthoparamyxovirinae* subfamily) and include some of the most pathogenic zoonotic viruses known to infect humans. The genus *Henipavirus* currently includes several recognized species, based on International Committee on Taxonomy of Viruses classification: *Henipavirus hendraense* (commonly referred to as Hendra virus, HeV), *nipahense* (Nipah virus, NiV), *cedarense*, *ghanaense*, and *angavokelyense* (Walker et al., 2022). Since the initial emergence of HeV in Australia in 1994 (Murray et al., 1995b) and NiV in Malaysia in 1998 (Chua et al., 1999), several significant NiV outbreaks have been documented, including a single outbreak in Singapore in 1999 (linked to transmission from Malaysia) (Paton et al., 1999), a single outbreak in the Philippines in 2014 (Ching et al., 2015), and recurrent outbreaks in India and Bangladesh from 2001 to the present (Harcourt et al., 2005; Chadha et al., 2006; Arankalle et al., 2011; Lo et al., 2012; Islam et al., 2016; Thomas et al., 2019; Yadav et al., 2019; Rahman et al., 2021; Sudeep et al., 2021; Paul et al., 2023; World Health Organization, 2025) (**Supplementary Figure S1**). Henipaviruses are primarily hosted by natural reservoirs such as *Pteropus* bats (commonly known as fruit bats or flying foxes), shrews, and rodents, which serve as asymptomatic carriers (Mougari et al., 2022; Li et al., 2023). Current evidence indicates that NiV transmission to humans occurs through zoonotic spillover, often via contact with contaminated animal excretions or consumption of infected food, such as date palm sap contaminated by bat saliva (Tan et al., 2024). NiV has repeatedly demonstrated human-to-human transmission in close-contact settings, with variability by lineage and context; outbreak amplification often occurs in healthcare or household clusters (Hegde et al., 2024). Human HeV infection occurs almost exclusively through close contact with infected horses, particularly via exposure to their respiratory secretions and other bodily fluids. Horses are the established intermediate host in human infections and direct bat-to-human transmission has not been confirmed (Murray et al., 1995a).

Clinically, henipaviruses cause severe disease, with symptoms ranging from febrile neurological disease and acute respiratory distress to pneumonia and fatal encephalitis. Outcomes are frequently severe, with reported case fatality rates reaching up to 75% for NiV and 57% for HeV, subject to variation across outbreaks, healthcare settings, and diagnostic criteria (Chua et al., 1999; Eaton et al., 2006). NiV and HeV diagnosis relies mainly on real-time RT-PCR for detecting viral RNA in respiratory samples, blood, urine, or cerebrospinal fluid during acute illness (Mazzola and Kelly-Cirino, 2019; World Organization for Animal Health, 2022). Serological assays such as ELISA for IgM/IgG are used in later stages or retrospectively, with confirmatory neutralization tests performed at reference laboratories. In research or fatal cases, virus isolation and immunohistochemistry on tissue samples may also be applied. Emerging point-of-care platforms show promise for rapid detection, but most remain in experimental or validation phases (Pollak et al., 2022; Miao et al., 2023; van den Hurk et al., 2025).

There are no licensed human treatments or vaccines for henipaviruses; clinical management is supportive for severe respiratory and neurologic complications (US Centers for Disease Control and Prevention, 2024). Among antiviral agents, remdesivir confers protection in non-human primates when treatment is initiated early, but only partial protection when treatment is delayed (Lo et al., 2019; de Wit et al., 2023); favipiravir shows activity in hamsters (with reports ranging from complete to partial protection) (Dawes et al., 2018; Johnston et al., 2025), while human efficacy data are lacking and ribavirin evidence remains limited to observational reports (Levine et al., 2025). The monoclonal antibody m102.4 (anti-G) is safe and well-tolerated in Phase 1 and has supportive animal and compassionate-use data (Bossart et al., 2011; Playford et al., 2020). Vaccines are progressing – PHV02 (rVSV-NiV-G) completed Phase 1 and is moving toward Phase 2 in Bangladesh (Coalition for Epidemic Preparedness Innovations, 2025), while ChAdOx1-NiV received European Medicines Agency (EMA) PRIority MEdicines (PRIME) support in 2025 (Oxford Vaccine Group, 2025) – and, in a One Health measure, Australia’s Equivac HeV equine vaccine is licensed to protect horses and reduce spillover risk (Zoetis, 2022). Taken together, the lack of specific antiviral treatments or approved vaccines for humans underscores the threat of NiV infection. Due to its high mortality, potential for human-to-human spread, and absence of effective medical countermeasures, NiV is prioritized by the WHO for urgent research to prevent future epidemics (World Health Organization, 2024).

Since the initial outbreaks of NiV in Southeast Asia and HeV in Australia, several novel henipaviruses have subsequently been discovered across Asia, Europe, Africa, and the Americas (**Table 1**). The frequency of new henipavirus and henipa-like orthoparamyxoviruses discoveries in recent years, especially in China and Europe, reflects intensified surveillance of high-risk small mammals and increased access to metagenomic approaches (Vanmechelen et al., 2022; Chen et al., 2023; Horemans et al., 2023; Haring et al., 2024; Kuang et al., 2025). In contrast, the relative paucity of detections in other regions may reflect surveillance gaps rather than true biogeographic restriction (Sun et al., 2024). To date, more than twenty genetically distinct henipaviruses (species demarcation within *Henipavirus* and henipa-like orthoparamyxoviruses is based on phylogenetic divergence of complete large (L) protein amino acid sequences, with distinct species defined by branch length separation (~0.03 substitutions per site) in L protein–based trees (Rima et al., 2019)) have been reported across all continents except Antarctica, with nearly half of these identified in China (**Supplementary Figures S2**, **S3**). For instance, within the shrew- and rodent-borne genetic cluster, Mojiang virus (MojV) was temporally linked to a fatal pneumonia cluster among miners in Yunnan province; however, a causal role remains unproven (Wu et al., 2014). Langya virus (LayV) has been detected in febrile patients in Shandong and Henan provinces, with no deaths reported to date (Zhang et al., 2022). In addition, several henipaviruses are identified from the shrews (*Crocidura*, *Chodsigoa*, and *Suncus* genus) and rodents (*Apodemus* genus) hosts: Wenzhou shrew henipavirus 1, Wenzhou *Apodemus agrarius* henipavirus 1, Jingmen *Crocidura shantungensis* henipavirus 1 and 2, Wufeng *Crocidura attenuata* henipavirus 1, and Wufeng *Chodsigoa smithii* henipavirus 1 (Chen et al., 2023). Interestingly, two novel henipaviruses are detected for the first time from bats in the Yunnan province in southwestern China, bordering a number of Southeast Asian countries (Kuang et al., 2025). Designated as Yunnan bat henipavirus 1 and Yunnan bat henipavirus 2, these henipaviruses are grouped within the bat-borne cluster – with the Yunnan bat henipavirus 1 identified as the closest known relative of NiV and HeV (**Supplementary Figure S2**). These viruses are detected in the kidneys of *Rousettus leschenaultii*, or the fulvous fruit bats, which are relatively widespread across South and Southeast Asia that thrive in diverse habitats, such as tropical forests, caves, agricultural areas and even urban environments. Similar to other henipavirus lineages newly discovered in China, no human infections or seropositivity are reported to date, although the pathogenicity and spillover risks of these viruses remain unclear (**Table 1**).

**Table 1:** Overview of the genus *Henipavirus* and henipa-like orthoparamyxoviruses (family *Paramyxoviridae*, subfamily *Orthoparamyxovirinae*), including geographic origin and year of first identification, reservoir hosts, potential for human infection, clinical disease characteristics, and reported case fatality rates.

Genus Henipavirus	Country of first identification	Year	Hosts	Human infection	Disease characteristics	Case fatality rate	Genome size, base pair (GenBank accession number)	References
Hendra virus	Australia	1994	Bats (*Pteropus alecto, conspicillatus, poliocephalus, scapulatus*)	Yes	Acute respiratory distress, encephalitis	57%	18,234 (NC00190)	Murray et al., 1995b
Nipah virus	Malaysia	1998	Bats (*Pteropus vampyrus, hypomelanus, lylei, medius*)	Yes	Acute respiratory distress, encephalitis	40-75%	18,246 (NC00272)	Chua et al., 1999
Cedar virus	Australia	2009	Bats (*Pteropus alecto, poliocephalus*)	ND*	NR^†^	NR	18,162 (NC02535)	Marsh et al., 2012
Ghana virus	Ghana	2009	Bats (*Eidolon helvum*)	ND	NR	NR	18,530 (NC025256)	Drexler et al., 2009
Angavokely virus	Madagascar	2019	Bats (*Eidolon dupreanum*)	ND	NR	NR	16,740 (ON613535)	Madera et al., 2022

Henipa-like orthoparamyxoviruses	Country of first identification	Year	Hosts	Human infection	Disease characteristics	Case fatality rate	Genome size, base pair (GenBank accession number)	References (or GenBank accession number^‡^)
Mojiang virus	China	2012	Rodents (*Rattus flavipectus*)^≈^	Yes	Pneumonia	100% ^#^	18,406 (NC025352)	Wu et al., 2014
Langya virus	China	2018	Shrews (*Crocidura lasiura, shantungensis*)	Yes	Fever, cough, fatigue	No deaths	18,402 (OM101125)	Zhang et al., 2022
Yunnan bat henipavirus 1	China	2017	Bats (*Rousettus leschenaultii*)	ND	NR	NR	19,755 (PQ621839)	Kuang et al., 2025
Yunnan bat henipavirus 2	China	2017	Bats (*Rousettus leschenaultii*)	ND	NR	NR	17,723 (PQ621840)	Kuang et al., 2025
Wenzhou shrew henipavirus 1	China	Unknown	Shrews (*Suncus murinus*), rodents (*Apodemus agrarius*)	ND	NR	NR	18,426 (OQ715593)	Chen et al., 2023
Wenzhou *Apodemus agrarius* henipavirus 1	China	2016	Rodents (*Apodemus agrarius*)	ND	NR	NR	18,309 (MZ328275)	MZ328275
Jingmen *Crocidura shantungensis* henipavirus 1	China	2016	Shrews (*Crocidura shantungensis*)	ND	NR	NR	18,535 (OM030314)	OM030314
Jingmen *Crocidura shantungensis* henipavirus 2	China	2016	Shrews (*Crocidura shantungensis*)	ND	NR	NR	19,461 (OM030315)	OM030315
Wufeng *Crocidura attenuata* henipavirus 1	China	2016	Shrews (*Crocidura attenuata*)	ND	NR	NR	18,379 (OM030317)	OM030317
Wufeng *Chodsigoa smithii* henipavirus 1	China	2016	Shrews (*Chodsigoa smithii*)	ND	NR	NR	18,443 (OM030316)	OM030316
Daeryong virus	South Korea	2017	Shrews (*Crocidura shantungensis*)	ND	NR	NR	19,471 (MZ574409)	Lee et al., 2021
Gamak virus	South Korea	2017	Shrews (*Crocidura lasiura*)	ND	NR	NR	18,460 (MZ574407)	Lee et al., 2021
Melian virus	Guinea	2018	Shrews (*Crocidura grandiceps*)	ND	NR	NR	19,944 (OK623353)	Vanmechelen et al., 2022
Denwin virus	Belgium	2019	Shrews (*Crocidura russula*)	ND	NR	NR	19,740 (OR713883)	Vanmechelen et al., 2022
Ninorex virus	Belgium	2020	Shrews (*Sorex minutus*)	ND	NR	NR	19,158 (OQ438286)	Horemans et al., 2023
Hasua virus	Germany	2020	Shrews (*Crocidura suaveolens*)	ND	NR	NR	18,468 (OR713881)	Haring et al., 2024
Lechcodon virus	Germany	2021	Shrews (*Crocidura leucodon*)	ND	NR	NR	18,928 (OR713879)	Haring et al., 2024
Peixe-Boi virus	Brazil	2015	Marsupials (*Marmosa demerarae*)	ND	NR	NR	2,377 (MZ615319) ^§^	Hernandez et al., 2022
Camp Hill virus	United States	2021	Shrews (*Blarina brevicauda*)	ND	NR	NR	16,681 (PQ140948)	Parry et al., 2025

^*^ ND, not detected (no evidence to date).

^†^ NR, not reported.

^‡^ The GenBank accession number is provided where published references are unavailable.

^≈^*Rattus flavipectus* is treated as a synonym of *R. tanezumi* in the IUCN Red List.

^#^ Based on limited case data and inconclusive.

^§^ Represented by a partial genome sequence (RNA-dependent RNA polymerase gene (RdRp); ~34% of the L gene, ~13% of the genome), which supports its classification as a henipa-like orthoparamyxovirus based on large (L) protein phylogeny, conserved RdRp motifs, and amino acid divergence patterns.

The recent detections of novel henipaviruses emphasize the critical need for comprehensive disease surveillance across human, animal, and environmental sectors. These findings raise concerns about emerging threats from previously undetected henipavirus lineages. With their potential capacity for cross-species transmission, such viruses pose a serious risk of future outbreaks. Strengthened surveillance, research, and regional as well as international coordination are essential to address these evolving threats.

## Overarching challenges & research priorities

### Integrated surveillance

One of the most pressing challenges in responding to the emergence of novel henipaviruses is the widespread lack of integrated genomic and serological surveillance systems. In many low- and middle-income countries (LMICs), particularly across Asia where henipavirus outbreaks have occurred and in parts of Africa where henipaviruses or their natural hosts have been identified (**Supplementary Figures S1**, **S3**), surveillance remains limited or narrowly focused on known pathogens, leaving divergent henipavirus strains undetected. For instance, WHO reported that one in three countries (about 33%) do not have in-country genomic surveillance capacity for pathogens (World Health Organization, 2022), despite the major investments made in genomic surveillance in response to the COVID-19 pandemic. Even fewer countries have established comprehensive serological monitoring that spans human, animal, and wildlife species (Schulz et al., 2020; Akoi Bore et al., 2024). Moreover, most surveillance efforts are disease-specific and fail to capture the broader virome present in wildlife or the environment. This fragmented approach hampers early warning capabilities, delaying outbreak detection and impeding rapid response, as seen in some previous spillover events of NiV where rapid and early case identification was not available (Hassan et al., 2025). Similarly, the identification of MojV as the etiological agent for fatal cases of pneumonia was established six months after the initial outbreak that occurred in an abandoned mine in Yunnan province (Wu et al., 2014).

To close critical knowledge and preparedness gaps related to novel and under-characterized henipavirus species, there is an urgent need to strengthen surveillance systems across both animal and human health domains (**Supplementary Figure S4**). A top priority is the expansion of genomic and serological surveillance in wildlife reservoirs, particularly bats, shrews, and rodents that have been identified as natural hosts or potential carriers, with coordinated sampling in human populations living in at-risk areas (Moore et al., 2024). To improve efficiency and impact, surveillance should be geographically targeted using spatial risk mapping that integrates ecological and environmental determinants of spillover risk, such as land-use change, climate variability, and host species distribution (Sun et al., 2024). By aligning surveillance activities with areas where cross-species transmission is most likely, data can be generated to identify high-risk interfaces (e.g., bat-livestock-human contact zones, wildlife markets) and to guide preventive interventions. Standardized protocols for specimen collection (e.g., blood, oral swabs, urine, feces) and sequencing workflows are needed to ensure both the quality and comparability of datasets across studies and regions. Strengthening surveillance also requires clear data governance frameworks to ensure timely but responsible sharing of genomic and epidemiological data across sectors and borders. Equally important are enabling conditions for safe wildlife sampling practices that emphasize personal protective equipment, cold chain maintenance, and biosafety standards.

Within this broader framework, serological surveillance plays a particularly important role in detecting past exposure and silent transmission, especially in the absence of active infection (**Supplementary Figure S4**) (Wang et al., 2023). Currently, assays are available only for NiV, HeV, LayV, and Cedar virus (largely limited to in-house or research-use platforms, as no commercialized diagnostic kits are available), but interpretation remains complicated by cross-reactivity with other paramyxoviruses. Using more specific antigens (e.g., glycoprotein or prefusion proteins) together with a tiered approach, such as ELISA or bead-based multiplex assays screening followed by neutralization assays, is recommended (Fischer et al., 2018). Such approaches not only enhance diagnostic accuracy but also provide more reliable data for understanding the true extent of henipavirus circulation in humans and animal reservoirs.

In tandem with environmental and animal surveillance, there is a critical need to establish longitudinal human cohort studies focusing on patients presenting with febrile, respiratory and neurological syndromes – clinical manifestations that may signal early or atypical henipavirus infections. Continuous clinical surveillance and systematic biobanking of patient samples in endemic or high-risk areas can facilitate the detection of emerging or cryptic viral threats. Integrating these patient-based cohorts with genomic diagnostics, metagenomic sequencing, and syndromic surveillance frameworks will enhance early outbreak detection and allow for better characterization of clinical spectrum and disease burden. Such cohort-based studies are especially important in resource-limited settings, where underdiagnosis is common and disease etiology often remains unresolved. Case surveillance studies have shown an alarmingly high frequency of febrile illnesses (~60%) (Rainey et al., 2022) and acute meningitis and encephalitis (~70%) (Wang et al., 2022) of unknown etiology, signaling under-detection of known and possibly novel pathogens, and point to systemic blind spots in diagnostic capacity and disease surveillance. Expanding access to syndromic and genomic surveillance will be essential to closing these gaps. Strengthening this clinical surveillance pillar alongside environmental and animal health monitoring is essential to building a comprehensive, real-time early warning system for henipaviruses (**Supplementary Figure S4**).

In the regional context of disease surveillance in China, sustained investment in laboratory capacity, including next-generation sequencing and biospecimen repositories, is critical for rapid detection and long-term monitoring. Yunnan province, where MojV and Yunnan bat henipaviruses 1 and 2 have been detected, is particularly vulnerable to cross-species transmission due to its exceptional biodiversity. The Yunnan Provincial Center for Disease Control and Prevention (CDC), for example, has been central to surveillance and genetic characterization of novel henipaviruses, bat coronaviruses, and other high-risk zoonoses in southern China (Luo et al., 2018; Yang et al., 2023; Kuang et al., 2025). Its continuous field sampling and collaborative virus discovery efforts have contributed valuable genomic data to global early warning systems.

At the policy level, cross-border surveillance networks, capacity building, and real-time data sharing are critical to accelerate recognition of emerging threats and enable timely public health responses. Because southern China and mainland Southeast Asia are linked by ecologically connected, transboundary habitats, the risk of henipavirus spillover extends across rather than within national boundaries. Yunnan province, which borders Myanmar, Laos, and Vietnam, is a region of particular concern. Strengthening cross-border collaboration is therefore essential. The Mekong Basin Disease Surveillance (MBDS) network has facilitated joint outbreak investigations, data governance and sharing, and workforce training since the early 2000s (Mekong Basin Disease Surveillance (MBDS) network, 2025), and renewed post-COVID-19 momentum offers an opportunity to reinvigorate this platform with a focus on emerging zoonoses. Likewise, the Association of Southeast Asian Nations (ASEAN)-China Health Cooperation has advanced diagnostics, surveillance, and preparedness through regional dialogue and technical collaboration (ASEAN-China Health Cooperation, 2024). Proactive inclusion of henipaviruses within these health security agendas is both timely and necessary, and we propose establishing a joint framework that bridges ASEAN-China platforms with Bangladesh and India to strengthen preparedness across the wider Asian corridor of risk.

### One Health implementation

**Figure 1:**
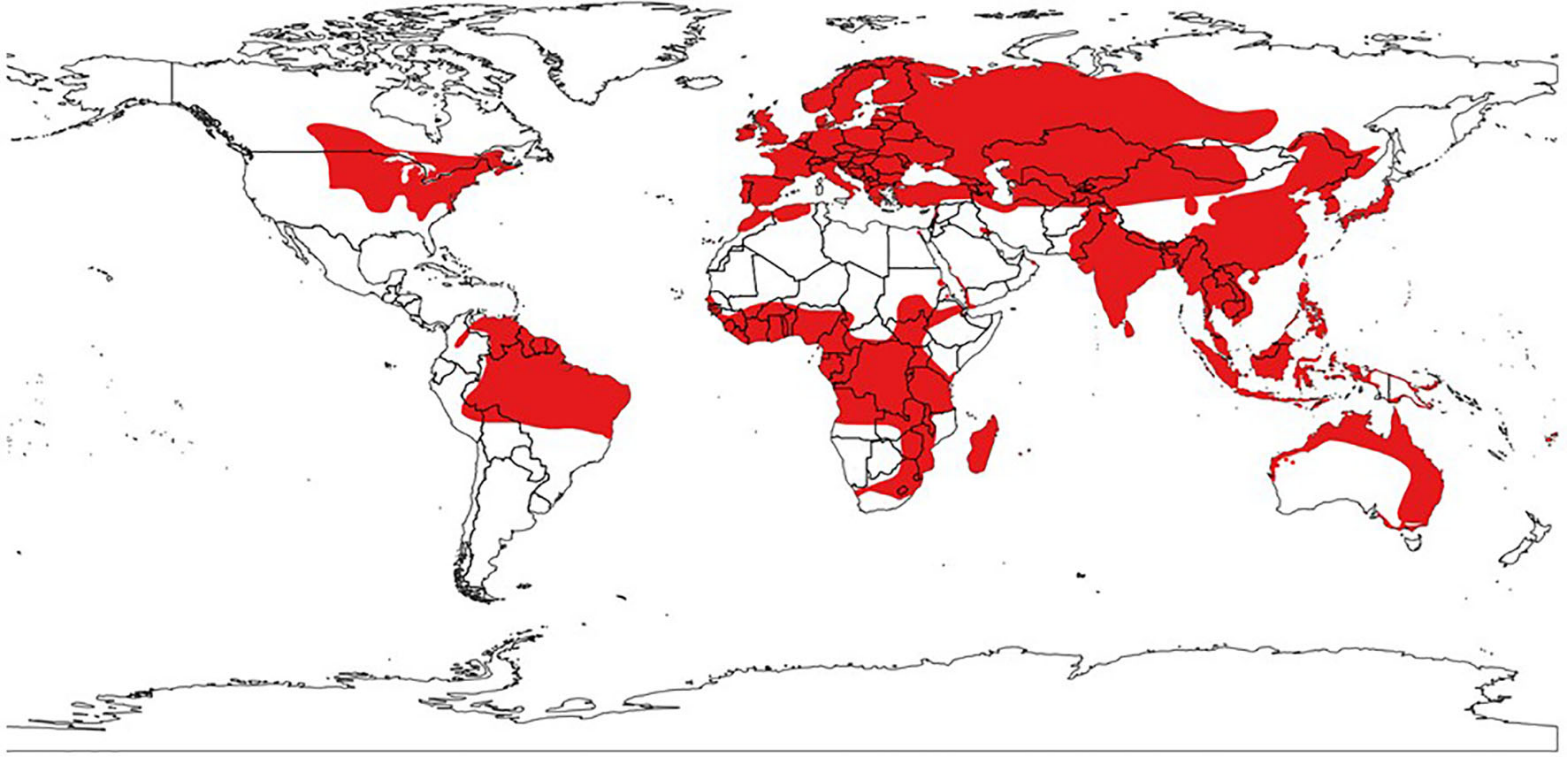
Global distribution of animal hosts of henipaviruses and related henipa-like orthoparamyxoviruses. Mapped regions (in red) indicate the ranges of all known natural hosts of henipaviruses and related henipa-like orthoparamyxoviruses (**Table 1**), including bats (*Pteropus*, *Eidolon*, *Rousettus* spp.), shrews (*Crocidura*, *Chodsigoa*, *Suncus*, *Sorex*, *Blarina* spp.), rodents (*Rattus*, *Apodemus* spp.), and marsupials (*Marmosa* spp.). Host range data were obtained from the International Union for Conservation of Nature (IUCN) Red List of Threatened Species (version 2025-1). Countries and territories overlapped with these ranges were identified through a vector–raster overlay analysis, using global administrative boundaries from the Natural Earth dataset (version 5.1.1); a unit was classified as included if at least 1%, 5% or 10% of its land area intersected a host range. This provides an approximate but reproducible method, with minor uncertainty related to resolution and classification thresholds.

Despite growing global recognition of the One Health approach, its implementation remains fragmented and inconsistent across countries and sectors. Human, animal, and environmental health sectors often continue to function independently, hindering data sharing and joint risk assessment. The natural hosts of henipaviruses and related henipa-like orthoparamyxoviruses are now documented in more than 130 countries and territories (**Figure 1**; **Supplementary Table S1**), underscoring a wider potential for spillover than previously recognized. However, geographic presence does not equate to local abundance, and neither distribution nor density alone determines spillover risk, which is shaped by additional ecological and anthropogenic factors. Critical interfaces such as those between wildlife, livestock, and humans remain under-studied, particularly in rural and peri-urban settings where risk is likely elevated. A recent global assessment reported that formal coordination mechanisms across One Health sectors are underdeveloped in many LMICs (Zhang et al., 2024). Additionally, ecosystem-level drivers of disease emergence, including deforestation, agricultural intensification, and biodiversity loss, are rarely monitored in tandem with health surveillance systems. Southeast Asia, a known hotspot for zoonotic spillovers, has experienced rapid land-use changes, with over 60 million hectares of forest lost in the last two decades (Feng et al., 2021), intensifying contact between wildlife and human populations (Sanchez et al., 2022). Without stronger intersectoral coordination and environmental monitoring, the world remains vulnerable to future henipavirus outbreaks and other zoonotic threats.

Effective implementation of the One Health approach for henipavirus prevention requires a research agenda that addresses critical gaps in transmission ecology and environmental risk. One priority area is the characterization of viral shedding dynamics in reservoir and intermediate hosts, particularly bats, shrews, and livestock species. Longitudinal surveillance studies that monitor viral shedding in host urine across different seasons and physiological states (e.g., breeding, gestation, or migration) could help identify temporal patterns associated with increased zoonotic risk (Plowright et al., 2015). Such data, when coupled with ecological and climatic variables, would enable more accurate modeling of spillover risk and inform targeted interventions at high-risk interfaces.

Spatial risk models that integrate remote sensing data, species distribution records, and land-use change metrics offer a promising tool for predicting potential hotspots of viral emergence (Plowright et al., 2021; Carlson et al., 2022). By combining satellite-derived environmental indicators (e.g., vegetation cover, rainfall anomalies, temperature shifts) with high-resolution maps of host and reservoir species distributions, these models can capture ecological conditions that facilitate viral maintenance and transmission. Incorporating land-use dynamics, such as deforestation, agricultural expansion, and urban encroachment, further helps identify areas where human activities increase contact with wildlife reservoirs (Jones et al., 2013). Operationalizing these insights requires a dashboard of environmental drivers that integrates deforestation alerts, livestock density data, and culturally relevant risk practices (e.g., unprotected date palm sap harvesting) into a public, quarterly updated platform. Such a tool enables real-time monitoring of spillover drivers and issues timely advisories to national and regional stakeholders. Measurable outputs include the public availability of the dashboard, the number of risk advisories generated, and documented evidence of their uptake in preparedness actions. Together, these integrated approaches can pinpoint geographic zones of elevated spillover risk and support scenario-based forecasting, providing evidence to guide targeted surveillance, early warning systems, and preventive interventions (**Supplementary Figure S4**).

### Diagnostic infrastructure

Diagnostic capacity for henipaviruses remains severely constrained, with testing confined to a limited number of specialized reference laboratories equipped with advanced biosafety facilities (World Health Organization, 2022). This gap represents a critical barrier to timely outbreak detection and case confirmation, especially in regions at highest risk of zoonotic spillover. A recent survey highlighted that fewer than 20% of health facilities in low-income settings, particularly across Africa, had access to diagnostic platforms, even for high-burden diseases such as HIV/AIDS and malaria (Yadav et al., 2021), underscoring the magnitude of inequities in laboratory capacity. Within this context, point-of-care assays and rapid diagnostic tools for henipaviruses remain scarce, especially in rural and high-risk regions where they are most needed (Hassan et al., 2025).

Addressing these diagnostic gaps requires urgent investment in both tool development and system-level strengthening (Okeke and Ihekweazu, 2021). A priority is the development and validation of multiplex PCR assays and rapid antigen tests capable of detecting a broad range of known and novel henipaviruses, enabling differential diagnosis in febrile, respiratory, and encephalitic illness presentations. While metagenomic sequencing offers unparalleled breadth for pathogen discovery, its implementation in low-resource settings is constrained by cost, infrastructure requirements, and technical complexity, making multiplex PCR a more feasible frontline solution. Equally critical is the establishment of global validation pipelines, such as WHO prequalification and regional reference standards, to ensure diagnostic accuracy and comparability across platforms.

Emerging point-of-care platforms such as reverse transcription loop-mediated isothermal amplification (RT-LAMP), reverse transcription recombinase polymerase amplification (RT-RPA), and CRISPR-based assays offer promising alternatives for rapid detection of NiV and HeV (Pollak et al., 2022; Miao et al., 2023; van den Hurk et al., 2025). Compared with multiplex PCR, these methods are generally faster, easier to use, and less reliant on advanced laboratory infrastructure, which makes them attractive for deployment in resource-limited settings (**Supplementary Table S2**). They also have the potential to reduce costs and cold-chain dependence, although reagent stability in tropical environments remains a challenge. CRISPR-based assays, in particular, offer high specificity through programmable guide RNAs, but like other isothermal methods, they remain in early development or validation with limited performance data, constraining their immediate application for large-scale surveillance or outbreak response. It is also important to note that primer and probe design for henipavirus assays requires particular attention to lineage divergence within the G, N, and F genes. For broad coverage, degenerate primers or pooled primer sets are often necessary, complemented by *in silico* inclusivity analyses against all available NiV and HeV sequences. Such analyses are essential to ensure that assays can reliably detect both prototype and divergent strains, and to minimize false negatives when applied in surveillance across regions where multiple lineages co-circulate. Therefore, while these assays represent promising complements to multiplex PCR, significant hurdles must be overcome before they can reliably support henipavirus surveillance and outbreak response. Accordingly, multiplex RT-PCR remains the primary clinical confirmatory method (Guillaume et al., 2004; Feldman et al., 2009; Mazzola and Kelly-Cirino, 2019), while isothermal and CRISPR-based assays are better positioned for field screening and surge triage pending further validation.

Expanding local laboratory capacity, especially in LMICs where spillover risk is concentrated, will require improving assay performance, strengthening regional networks, workforce training, and collaborative technology transfer. Together, these measures are essential to close the diagnostic divide and enhance early detection of henipavirus spillover events.

While genomic characterization and molecular diagnostics remain central to henipavirus detection and surveillance, it is important to note that functional virological assays are often required to assess host range and zoonotic potential of newly identified henipaviruses. In particular, pathogenic henipaviruses such as NiV and HeV are known to utilize the highly conserved ephrin-B2 and ephrin-B3 cellular receptors, a feature that underpins their broad mammalian tropism and severe pathogenicity (Bonaparte et al., 2005; Negrete et al., 2005). Accordingly, receptor-binding and pseudotyped virus entry assays, as well as reverse-genetics-based systems, have been widely employed to interrogate viral entry, fusion efficiency, and replication competence across species (Yun et al., 2015). Comprehensive descriptions of functional approaches for receptor usage studies and reverse-genetics platforms for henipaviruses have been reported elsewhere, and are not further discussed in this article, which focuses on molecular diagnostics and detection strategies applicable to surveillance and outbreak settings.

## Concluding remarks

The identification of novel henipaviruses and henipa-like orthoparamyxoviruses underscores the persistent risk of zoonotic spillover and the need for proactive global health measures. Current limitations in surveillance, fragmented One Health coordination, and inadequate diagnostic tools in high-risk regions leave the world vulnerable to outbreaks. Addressing these challenges requires a multifaceted approach: expanding genomic and serological surveillance in wildlife and human populations, fostering intersectoral collaboration under the One Health framework, and investing in accessible diagnostic technologies. By prioritizing these strategies, the global community can enhance early detection, improve outbreak response, and mitigate the threat of henipaviruses. As these viruses continue to evolve, sustained research, international cooperation, and equitable resource allocation will be essential to safeguarding public health against future epidemics.

## Data Availability

The original contributions presented in the study are included in the article/**Supplementary Material**. Further inquiries can be directed to the corresponding authors.

## References

[B1] Akoi Bore J. Timothy J. W. S. Tipton T. Kekoura I. Hall Y. Hood G. . (2024). Serological evidence of zoonotic filovirus exposure among bushmeat hunters in Guinea. Nat. Commun. 15, 4171. doi: 10.1038/s41467-024-48587-5, PMID: 38755147 PMC11099012

[B2] Arankalle V. A. Bandyopadhyay B. T. Ramdasi A. Y. Jadi R. Patil D. R. Rahman M. . (2011). Genomic characterization of Nipah virus, West Bengal, India. Emerg. Infect. Dis. 17, 907–909. doi: 10.3201/eid1705.100968, PMID: 21529409 PMC3321761

[B3] ASEAN-China Health Cooperation (2024). Joint statement of the ninth ASEAN China health ministers meeting (9th ACHMM). Available online at: https://asean.org/joint-statement-of-the-ninth-asean-China-health-ministers-meeting-9th-ac-hmm/ (Accessed July 20, 2025).

[B4] Bonaparte M. I. Dimitrov A. S. Bossart K. N. Crameri G. Mungall B. A. Bishop K. A. . (2005). Ephrin-B2 ligand is a functional receptor for Hendra virus and Nipah virus. Proc. Natl. Acad. Sci. U. S. A. 102, 10652–10657. doi: 10.1073/pnas.0504887102, PMID: 15998730 PMC1169237

[B5] Bossart K. N. Geisbert T. W. Feldmann H. Zhu Z. Feldmann F. Geisbert J. B. . (2011). A neutralizing human monoclonal antibody protects african green monkeys from hendra virus challenge. Sci. Transl. Med. 3, 105ra3. doi: 10.1126/scitranslmed.3002901, PMID: 22013123 PMC3313625

[B6] Carlson C. J. Albery G. F. Merow C. Trisos C. H. Zipfel C. M. Eskew E. A. . (2022). Climate change increases cross-species viral transmission risk. Nature 607, 555–562. doi: 10.1038/s41586-022-04788-w, PMID: 35483403

[B7] Chadha M. S. Comer J. A. Lowe L. Rota P. A. Rollin P. E. Bellini W. J. . (2006). Nipah virus-associated encephalitis outbreak, Siliguri, India. Emerg. Infect. Dis. 12, 235–240. doi: 10.3201/eid1202.051247, PMID: 16494748 PMC3373078

[B8] Chen Y. M. Hu S. J. Lin X. D. Tian J. H. Lv J. X. Wang M. R. . (2023). Host traits shape virome composition and virus transmission in wild small mammals. Cell 186, 4662–75 e12. doi: 10.1016/j.cell.2023.08.029, PMID: 37734372

[B9] Ching P. K. de los Reyes V. C. Sucaldito M. N. Tayag E. Columna-Vingno A. B. Malbas F. F. Jr. . (2015). Outbreak of henipavirus infection, Philippines, 2014. Emerg. Infect. Dis. 21, 328–331. doi: 10.3201/eid2102.141433, PMID: 25626011 PMC4313660

[B10] Chua K. B. Goh K. J. Wong K. T. Kamarulzaman A. Tan P. S. Ksiazek T. G. . (1999). Fatal encephalitis due to Nipah virus among pig-farmers in Malaysia. Lancet 354, 1257–1259. doi: 10.1016/S0140-6736(99)04299-3, PMID: 10520635

[B11] Coalition for Epidemic Preparedness Innovations (2025). New vaccine set for human trials in Nipah outbreak hotspot. Available online at: https://cepi.net/new-vaccine-set-human-trials-nipah-outbreak-hotspot (Accessed August 30, 2025).

[B12] Dawes B. E. Kalveram B. Ikegami T. Juelich T. Smith J. K. Zhang L. . (2018). Favipiravir (T-705) protects against Nipah virus infection in the hamster model. Sci. Rep. 8, 7604. doi: 10.1038/s41598-018-25780-3, PMID: 29765101 PMC5954062

[B13] de Wit E. Williamson B. N. Feldmann F. Goldin K. Lo M. K. Okumura A. . (2023). Late remdesivir treatment initiation partially protects African green monkeys from lethal Nipah virus infection. Antiviral Res. 216, 105658. doi: 10.1016/j.antiviral.2023.105658, PMID: 37356729 PMC10529221

[B14] Drexler J. F. Corman V. M. Gloza-Rausch F. Seebens A. Annan A. Ipsen A. . (2009). Henipavirus RNA in African bats. PloS One 4, e6367. doi: 10.1371/journal.pone.0006367, PMID: 19636378 PMC2712088

[B15] Eaton B. T. Broder C. C. Middleton D. Wang L. F. (2006). Hendra and Nipah viruses: different and dangerous. Nat. Rev. Microbiol. 4, 23–35. doi: 10.1038/nrmicro1323, PMID: 16357858 PMC7097447

[B16] Feldman K. S. Foord A. Heine H. G. Smith I. L. Boyd V. Marsh G. A. . (2009). Design and evaluation of consensus PCR assays for henipaviruses. J. Virol. Methods 161, 52–57. doi: 10.1016/j.jviromet.2009.05.014, PMID: 19477200

[B17] Feng Y. Ziegler A. D. Elsen P. R. Liu Y. He X. Spracklen D. V. . (2021). Upward expansion and acceleration of forest clearance in the mountains of Southeast Asia. Nat. Sustain. 4, 892–899. doi: 10.1038/s41893-021-00738-y

[B18] Fischer K. Diederich S. Smith G. Reiche S. Pinho Dos Reis V. Stroh E. . (2018). Indirect ELISA based on Hendra and Nipah virus proteins for the detection of henipavirus specific antibodies in pigs. PloS One 13, e0194385. doi: 10.1371/journal.pone.0194385, PMID: 29708971 PMC5927399

[B19] Guillaume V. Lefeuvre A. Faure C. Marianneau P. Buckland R. Lam S. K. . (2004). Specific detection of Nipah virus using real-time RT-PCR (TaqMan). J. Virol. Methods 120, 229–237. doi: 10.1016/j.jviromet.2004.05.018, PMID: 15288966

[B20] Harcourt B. H. Lowe L. Tamin A. Liu X. Bankamp B. Bowden N. . (2005). Genetic characterization of Nipah virus, Bangladesh, 2004. Emerg. Infect. Dis. 11, 1594–1597. doi: 10.3201/eid1110.050513, PMID: 16318702 PMC3366751

[B21] Haring V. C. Litz B. Jacob J. Brecht M. Bauswein M. Sehl-Ewert J. . (2024). Detection of novel orthoparamyxoviruses, orthonairoviruses and an orthohepevirus in European white-toothed shrews. Microb. Genom. 10, 001275. doi: 10.1099/mgen.0.001275, PMID: 39088249 PMC11293873

[B22] Hassan M. Z. Rojek A. Olliaro P. Horby P. (2025). Improving clinical care of patients in Nipah outbreaks: moving beyond ‘compassionate use’. Lancet Reg. Health Southeast Asia. 33, 100527. doi: 10.1016/j.lansea.2024.100527, PMID: 39866590 PMC11755010

[B23] Hegde S. T. Lee K. H. Styczynski A. Jones F. K. Gomes I. Das P. . (2024). Potential for person-to-person transmission of henipaviruses: A systematic review of the literature. J. Infect. Dis. 229, 733–742. doi: 10.1093/infdis/jiad467, PMID: 37925626 PMC10938219

[B24] Hernandez L. H. A. da Paz T. Y. B. Silva S. P. D. Silva F. S. D. Barros B. C. V. Nunes B. T. D. . (2022). First genomic evidence of a henipa-like virus in Brazil. Viruses 14, 2167. doi: 10.3390/v14102167, PMID: 36298723 PMC9608811

[B25] Horemans M. Van Bets J. Joly Maes T. Maes P. Vanmechelen B. (2023). Discovery and genome characterization of six new orthoparamyxoviruses in small Belgian mammals. Virus Evol. 9, vead065. doi: 10.1093/ve/vead065, PMID: 38034864 PMC10684267

[B26] Islam M. S. Sazzad H. M. Satter S. M. Sultana S. Hossain M. J. Hasan M. . (2016). Nipah virus transmission from bats to humans associated with drinking traditional liquor made from date palm sap, Bangladesh, 2011-2014. Emerg. Infect. Dis. 22, 664–670. doi: 10.3201/eid2204.151747, PMID: 26981928 PMC4806957

[B27] Johnston S. C. Qiu J. Norris S. L. W. Panchal R. Punger E. M. Teague M. . (2025). Dose response comparison of Nipah virus strains Malaysia and Bangladesh in hamsters exposed by the intranasal or intraperitoneal route. PloS One 20, e0318912. doi: 10.1371/journal.pone.0318912, PMID: 40354368 PMC12068590

[B28] Jones B. A. Grace D. Kock R. Alonso S. Rushton J. Said M. Y. . (2013). Zoonosis emergence linked to agricultural intensification and environmental change. Proc. Natl. Acad. Sci. U. S. A. 110, 8399–8404. doi: 10.1073/pnas.1208059110, PMID: 23671097 PMC3666729

[B29] Kuang G. Yang T. Yang W. Wang J. Pan H. Pan Y. . (2025). Infectome analysis of bat kidneys from Yunnan province, China, reveals novel henipaviruses related to Hendra and Nipah viruses and prevalent bacterial and eukaryotic microbes. PloS Pathog. 21, e1013235. doi: 10.1371/journal.ppat.1013235, PMID: 40554741 PMC12187171

[B30] Lee S. H. Kim K. Kim J. No J. S. Park K. Budhathoki S. . (2021). Discovery and genetic characterization of novel paramyxoviruses related to the genus henipavirus in crocidura species in the Republic of Korea. Viruses 13, 2020. doi: 10.3390/v13102020, PMID: 34696450 PMC8537881

[B31] Levine C. B. Sauer L. M. McLellan S. L. F. Evans J. D. State of the Science Working Group of the National Ebola T, Education Center’s Special Pathogens Research N (2025). Nipah virus: a summary for clinicians. Int. J. Emerg. Med. 18, 126. doi: 10.1186/s12245-025-00916-1, PMID: 40634915 PMC12239417

[B32] Li H. Kim J. V. Pickering B. S. (2023). Henipavirus zoonosis: outbreaks, animal hosts and potential new emergence. Front. Microbiol. 14, 1167085. doi: 10.3389/fmicb.2023.1167085, PMID: 37529329 PMC10387552

[B33] Lo M. K. Feldmann F. Gary J. M. Jordan R. Bannister R. Cronin J. . (2019). Remdesivir (GS-5734) protects African green monkeys from Nipah virus challenge. Sci. Transl. Med. 11, eaau9242. doi: 10.1126/scitranslmed.aau9242, PMID: 31142680 PMC6732787

[B34] Lo M. K. Lowe L. Hummel K. B. Sazzad H. M. Gurley E. S. Hossain M. J. . (2012). Characterization of Nipah virus from outbreaks in Bangladesh, 2008-2010. Emerg. Infect. Dis. 18, 248–255. doi: 10.3201/eid1802.111492, PMID: 22304936 PMC3310473

[B35] Luo Y. Li B. Jiang R. D. Hu B. J. Luo D. S. Zhu G. J. . (2018). Longitudinal surveillance of betacoronaviruses in fruit bats in Yunnan Province, China during 2009-2016. Virol. Sin. 33, 87–95. doi: 10.1007/s12250-018-0017-2, PMID: 29500692 PMC6178081

[B36] Madera S. Kistler A. Ranaivoson H. C. Ahyong V. Andrianiaina A. Andry S. . (2022). Discovery and genomic characterization of a novel henipavirus, angavokely virus, from fruit bats in Madagascar. J. Virol. 96, e0092122. doi: 10.1128/jvi.00921-22, PMID: 36040175 PMC9517717

[B37] Marsh G. A. de Jong C. Barr J. A. Tachedjian M. Smith C. Middleton D. . (2012). Cedar virus: a novel Henipavirus isolated from Australian bats. PloS Pathog. 8, e1002836. doi: 10.1371/journal.ppat.1002836, PMID: 22879820 PMC3410871

[B38] Mazzola L. T. Kelly-Cirino C. (2019). Diagnostics for Nipah virus: a zoonotic pathogen endemic to Southeast Asia. BMJ Glob. Health 4, e001118. doi: 10.1136/bmjgh-2018-001118, PMID: 30815286 PMC6361328

[B39] Mekong Basin Disease Surveillance (MBDS) network (2025). Mekong Basin Disease Surveillance (MBDS) network. Available online at: https://www.mbdsnet.org/about-us/ (Accessed July 18, 2025).

[B40] Miao J. Zuo L. He D. Fang Z. Berthet N. Yu C. . (2023). Rapid detection of Nipah virus using the one-pot RPA-CRISPR/Cas13a assay. Virus Res. 332, 199130. doi: 10.1016/j.virusres.2023.199130, PMID: 37178792 PMC10345719

[B41] Moore K. A. Mehr A. J. Ostrowsky J. T. Ulrich A. K. Moua N. M. Fay P. C. . (2024). Measures to prevent and treat Nipah virus disease: research priorities for 2024-29. Lancet Infect. Dis. 24, e707–ee17. doi: 10.1016/S1473-3099(24)00262-7, PMID: 38964362

[B42] Mougari S. Gonzalez C. Reynard O. Horvat B. (2022). Fruit bats as natural reservoir of highly pathogenic henipaviruses: balance between antiviral defense and viral toleranceInteractions between Henipaviruses and their natural host, fruit bats. Curr. Opin. Virol. 54, 101228. doi: 10.1016/j.coviro.2022.101228, PMID: 35533525

[B43] Murray K. Rogers R. Selvey L. Selleck P. Hyatt A. Gould A. . (1995a). A novel morbillivirus pneumonia of horses and its transmission to humans. Emerg. Infect. Dis. 1, 31–33. doi: 10.3201/eid0101.950107, PMID: 8903153 PMC2626820

[B44] Murray K. Selleck P. Hooper P. Hyatt A. Gould A. Gleeson L. . (1995b). A morbillivirus that caused fatal disease in horses and humans. Science 268, 94–97. doi: 10.1126/science.7701348, PMID: 7701348

[B45] Negrete O. A. Levroney E. L. Aguilar H. C. Bertolotti-Ciarlet A. Nazarian R. Tajyar S. . (2005). EphrinB2 is the entry receptor for Nipah virus, an emergent deadly paramyxovirus. Nature 436, 401–405. doi: 10.1038/nature03838, PMID: 16007075

[B46] Okeke I. N. Ihekweazu C. (2021). The importance of molecular diagnostics for infectious diseases in low-resource settings. Nat. Rev. Microbiol. 19, 547–548. doi: 10.1038/s41579-021-00598-5, PMID: 34183821 PMC8237771

[B47] Oxford Vaccine Group (2025). Oxford vaccine against deadly Nipah virus granted EMA PRIME designation for the first time. Available online at: https://www.ovg.ox.ac.uk/news/oxford-vaccine-against-deadly-nipah-virus-granted-ema-prime-designation-for-the-first-time (Accessed July 3, 2025).

[B48] Parry R. H. Yamada K. Y. H. Hood W. R. Zhao Y. Lu J. Y. Seluanov A. . (2025). Henipavirus in northern short-tailed shrew, Alabama, USA. Emerg. Infect. Dis. 31, 392–394. doi: 10.3201/eid3102.241155, PMID: 39983689 PMC11845136

[B49] Paton N. I. Leo Y. S. Zaki S. R. Auchus A. P. Lee K. E. Ling A. E. . (1999). Outbreak of Nipah-virus infection among abattoir workers in Singapore. Lancet 354, 1253–1256. doi: 10.1016/S0140-6736(99)04379-2, PMID: 10520634

[B50] Paul D. Mohanty A. Shah A. Kumar Padhi B. Sah R. (2023). Outbreak of an emerging zoonotic Nipah virus: An emerging concern. J. Biosaf. Biosecur. 5, 57–59. doi: 10.1016/j.jobb.2023.04.002, PMID: 37131986 PMC10127665

[B51] Playford E. G. Munro T. Mahler S. M. Elliott S. Gerometta M. Hoger K. L. . (2020). Safety, tolerability, pharmacokinetics, and immunogenicity of a human monoclonal antibody targeting the G glycoprotein of henipaviruses in healthy adults: a first-in-human, randomised, controlled, phase 1 study. Lancet Infect. Dis. 20, 445–454. doi: 10.1016/S1473-3099(19)30634-6, PMID: 32027842

[B52] Plowright R. K. Eby P. Hudson P. J. Smith I. L. Westcott D. Bryden W. L. . (2015). Ecological dynamics of emerging bat virus spillover. Proc. Biol. Sci. 282, 20142124. doi: 10.1098/rspb.2014.2124, PMID: 25392474 PMC4262174

[B53] Plowright R. K. Reaser J. K. Locke H. Woodley S. J. Patz J. A. Becker D. J. . (2021). Land use-induced spillover: a call to action to safeguard environmental, animal, and human health. Lancet Planet Health 5, e237–ee45. doi: 10.1016/S2542-5196(21)00031-0, PMID: 33684341 PMC7935684

[B54] Pollak N. M. Olsson M. Marsh G. A. Macdonald J. McMillan D. (2022). Evaluation of three rapid low-resource molecular tests for Nipah virus. Front. Microbiol. 13, 1101914. doi: 10.3389/fmicb.2022.1101914, PMID: 36845977 PMC9949527

[B55] Rahman M. Z. Islam M. M. Hossain M. E. Rahman M. M. Islam A. Siddika A. . (2021). Genetic diversity of Nipah virus in Bangladesh. Int. J. Infect. Dis. 102, 144–151. doi: 10.1016/j.ijid.2020.10.041, PMID: 33129964

[B56] Rainey J. J. Siesel C. Guo X. Yi L. Zhang Y. Wu S. . (2022). Etiology of acute febrile illnesses in Southern China: Findings from a two-year sentinel surveillance project, 2017-2019. PloS One 17, e0270586. doi: 10.1371/journal.pone.0270586, PMID: 35763515 PMC9239456

[B57] Rima B. Balkema-Buschmann A. Dundon W. G. Duprex P. Easton A. Fouchier R. . (2019). ICTV virus taxonomy profile: paramyxoviridae. J. Gen. Virol. 100, 1593–1594. doi: 10.1099/jgv.0.001328, PMID: 31609197 PMC7273325

[B58] Sanchez C. A. Li H. Phelps K. L. Zambrana-Torrelio C. Wang L. F. Zhou P. . (2022). A strategy to assess spillover risk of bat SARS-related coronaviruses in Southeast Asia. Nat. Commun. 13, 4380. doi: 10.1038/s41467-022-31860-w, PMID: 35945197 PMC9363439

[B59] Schulz J. E. Seifert S. N. Thompson J. T. Avanzato V. Sterling S. L. Yan L. . (2020). Serological evidence for henipa-like and filo-like viruses in Trinidad bats. J. Infect. Dis. 221, S375–SS82. doi: 10.1093/infdis/jiz648, PMID: 32034942 PMC7213578

[B60] Sudeep A. B. Yadav P. D. Gokhale M. D. Balasubramanian R. Gupta N. Shete A. . (2021). Detection of Nipah virus in Pteropus medius in 2019 outbreak from Ernakulam district, Kerala, India. BMC Infect. Dis. 21, 162. doi: 10.1186/s12879-021-05865-7, PMID: 33563231 PMC7871573

[B61] Sun Y. Q. Zhang Y. Y. Liu M. C. Chen J. J. Li T. T. Liu Y. N. . (2024). Mapping the distribution of Nipah virus infections: a geospatial modelling analysis. Lancet Planet Health 8, e463–ee75. doi: 10.1016/S2542-5196(24)00119-0, PMID: 38969474 PMC12513742

[B62] Tan F. H. Sukri A. Idris N. Ong K. C. Schee J. P. Tan C. T. . (2024). A systematic review on Nipah virus: global molecular epidemiology and medical countermeasures development. Virus Evol. 10, veae048. doi: 10.1093/ve/veae048, PMID: 39119137 PMC11306115

[B63] Thomas B. Chandran P. Lilabi M. P. George B. Sivakumar C. P. Jayadev V. K. . (2019). Nipah virus infection in Kozhikode, Kerala, South India, in 2018: epidemiology of an outbreak of an emerging disease. Indian J. Community Med. 44, 383–387. doi: 10.4103/ijcm.IJCM_198_19, PMID: 31802805 PMC6881878

[B64] US Centers for Disease Control and Prevention (2024). Nipah virus: Facts for Clinicians. Available online at: https://www.cdc.gov/nipah-virus/hcp/clinical-overview/index.html (Accessed August 28, 2025).

[B65] van den Hurk S. Yondo A. Velayudhan B. T. (2025). Laboratory diagnosis of hendra and Nipah: two emerging zoonotic diseases with one health significance. Viruses 17, 1003. doi: 10.3390/v17071003, PMID: 40733619 PMC12299439

[B66] Vanmechelen B. Meurs S. Horemans M. Loosen A. Joly Maes T. Laenen L. . (2022). The characterization of multiple novel paramyxoviruses highlights the diverse nature of the subfamily Orthoparamyxovirinae. Virus Evol. 8, veac061. doi: 10.1093/ve/veac061, PMID: 35854826 PMC9290864

[B67] Walker P. J. Siddell S. G. Lefkowitz E. J. Mushegian A. R. Adriaenssens E. M. Alfenas-Zerbini P. . (2022). Recent changes to virus taxonomy ratified by the International Committee on Taxonomy of Viruses (2022). Arch. Virol. 167, 2429–2440. doi: 10.1007/s00705-022-05516-5, PMID: 35999326 PMC10088433

[B68] Wang L. F. Mani S. Tan C. W. Anderson D. E. (2023). Assays for detecting henipavirus antibodies. Methods Mol. Biol. 2682, 245–258. doi: 10.1007/978-1-0716-3283-3_18, PMID: 37610587

[B69] Wang L. P. Yuan Y. Liu Y. L. Lu Q. B. Shi L. S. Ren X. . (2022). Etiological and epidemiological features of acute meningitis or encephalitis in China: a nationwide active surveillance study. Lancet Reg. Health West Pac. 20, 100361. doi: 10.1016/j.lanwpc.2021.100361, PMID: 35036977 PMC8743210

[B70] World Health Organization (2022). Global genomic surveillance strategy for pathogens with pandemic and epidemic potential, 2022–2032 (Geneva: World Health Organization). 10.2471/BLT.22.288220 PMC8958828 35386562

[B71] World Health Organization (2024). Pathogens prioritization: a scientific framework for epidemic and pandemic research preparedness (Geneva: World Health Organization).

[B72] World Health Organization (2025). Nipah virus infection - India. Available online at: https://www.who.int/emergencies/disease-outbreak-news/item/2025-DON577 (Accessed August 31, 2025).

[B73] World Organization for Animal Health (2022). “ Nipah and hendra virus diseases,” in Manual of Diagnostic Tests and Vaccines for Terrestrial Animals (Paris, France: World Organization for Animal Health).

[B74] Wu Z. Yang L. Yang F. Ren X. Jiang J. Dong J. . (2014). Novel Henipa-like virus, Mojiang Paramyxovirus, in rats, China, 2012. Emerg. Infect. Dis. 20, 1064–1066. doi: 10.3201/eid2006.131022, PMID: 24865545 PMC4036791

[B75] Yadav H. Shah D. Sayed S. Horton S. Schroeder L. F. (2021). Availability of essential diagnostics in ten low-income and middle-income countries: results from national health facility surveys. Lancet Glob. Health 9, e1553–e1e60. doi: 10.1016/S2214-109X(21)00442-3, PMID: 34626546 PMC8526361

[B76] Yadav P. D. Shete A. M. Kumar G. A. Sarkale P. Sahay R. R. Radhakrishnan C. . (2019). Nipah virus sequences from humans and bats during Nipah outbreak, Kerala, India, 2018. Emerg. Infect. Dis. 25, 1003–1006. doi: 10.3201/eid2505.181076, PMID: 31002049 PMC6478210

[B77] Yang T. Yang W. Kuang G. Pan H. Han X. Yang L. . (2023). Prevalence and characteristics of novel pathogenic leptospira species in bats in Yunnan Province, China. Microorganisms 11, 1619. doi: 10.3390/microorganisms11061619, PMID: 37375121 PMC10304405

[B78] Yun T. Park A. Hill T. E. Pernet O. Beaty S. M. Juelich T. L. . (2015). Efficient reverse genetics reveals genetic determinants of budding and fusogenic differences between Nipah and Hendra viruses and enables real-time monitoring of viral spread in small animal models of henipavirus infection. J. Virol. 89, 1242–1253. doi: 10.1128/JVI.02583-14, PMID: 25392218 PMC4300668

[B79] Zhang X. A. Li H. Jiang F. C. Zhu F. Zhang Y. F. Chen J. J. . (2022). A zoonotic henipavirus in febrile patients in China. N Engl. J. Med. 387, 470–472. doi: 10.1056/NEJMc2202705, PMID: 35921459

[B80] Zhang Q. Liu J. Han L. Li X. Zhang C. Guo Z. . (2024). How far has the globe gone in achieving One Health? Current evidence and policy implications based on global One Health index. Sci. One Health 3, 100064., PMID: 39077388 10.1016/j.soh.2024.100064 PMC11262257

[B81] Zoetis (2022). Equivac HeV. Available online at: https://www.zoetis.com.au/all-products/portal-site/equivac-hev.aspx (Accessed July 3, 2025).

