# Supplementary material for: Strengthening preparedness and response to emerging henipavirus diversity
Source: Front Cell Infect Microbiol. 2026 Feb 25;16:1761347. doi: 10.3389/fcimb.2026.1761347 (PMC12975913; doi:10.3389/fcimb.2026.1761347)
Supplement: Supplementary file 1 [file DataSheet1.pdf]

Supplementary materials

Table of contents

**Materials**	**Description**	Page
Supplementary figure 1	Geographic distribution of Nipah virus, Hendra virus, and Nipah-like illness outbreaks, and the ranges of fruit bats of *Pteropodidae* family and *Pteropus* genus	2
Supplementary figure 2	Phylogenetic tree of nucleocapsid (N) protein amino acid sequences from henipaviruses and related henipa-like orthoparamyxoviruses, rooted with a *Jeilongvirus* sequence.	3
Supplementary figure 3	Henipaviruses and the geographical distribution of their animal hosts (listed in Table 1).	4
Supplementary figure 4	An integrated surveillance framework for Nipah virus, Hendra virus, and related henipa-like orthoparamyxoviruses, encompassing coordinated monitoring in humans, animals, and the environment.	5
Supplementary method 1	Spatial data sources, processing, threshold classification, and code availability.	6
Supplementary table 1	Countries and territories whose land area overlaps with the combined ranges of natural hosts of henipaviruses and related henipa-like orthoparamyxoviruses, evaluated at thresholds of ≥1%, ≥5%, and ≥10%.	7
Supplementary table 2	Comparative readiness of molecular platforms for henipavirus detection.	12

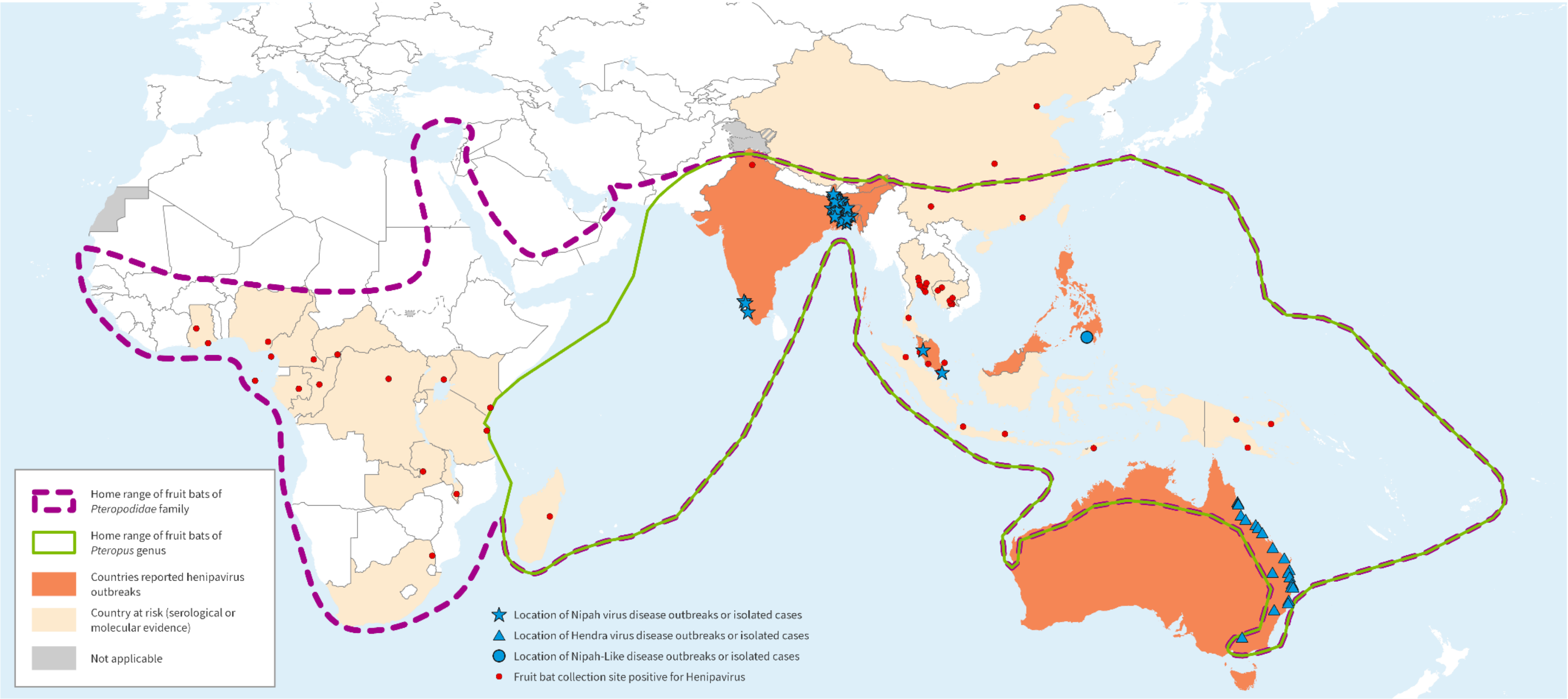

Supplementary figure 1. Geographic distribution of Nipah virus, Hendra virus, and Nipah-like illness outbreaks, and the ranges of fruit bats of *Pteropodidae* family and *Pteropus* genus. Figure adapted with permission from WHO (https://www.who.int/multi-media/details/geographic-distribution-of-henipavirus-outbreaks-and-fruit-bats-of-pteropodidae-family; accessed September 1, 2025).

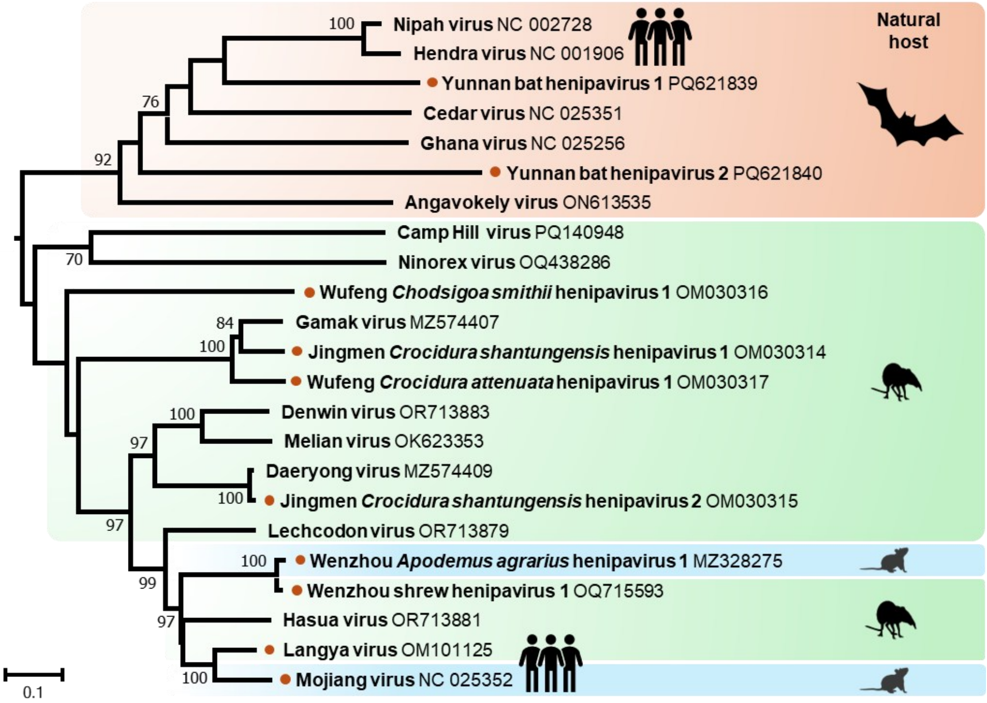

Supplementary figure 2. Phylogenetic tree of nucleocapsid (N) protein amino acid sequences from henipaviruses and related henipa-like orthoparamyxoviruses, rooted with a *Jeilongvirus* sequence. Coloured blocks denote host species: bats (brown), shrews (green), and rodents (blue). Newly identified viruses from China are highlighted with solid red circles. Strains known to infect humans (Nipah virus, Hendra virus, Langya virus, and Mojiang virus) are also indicated.

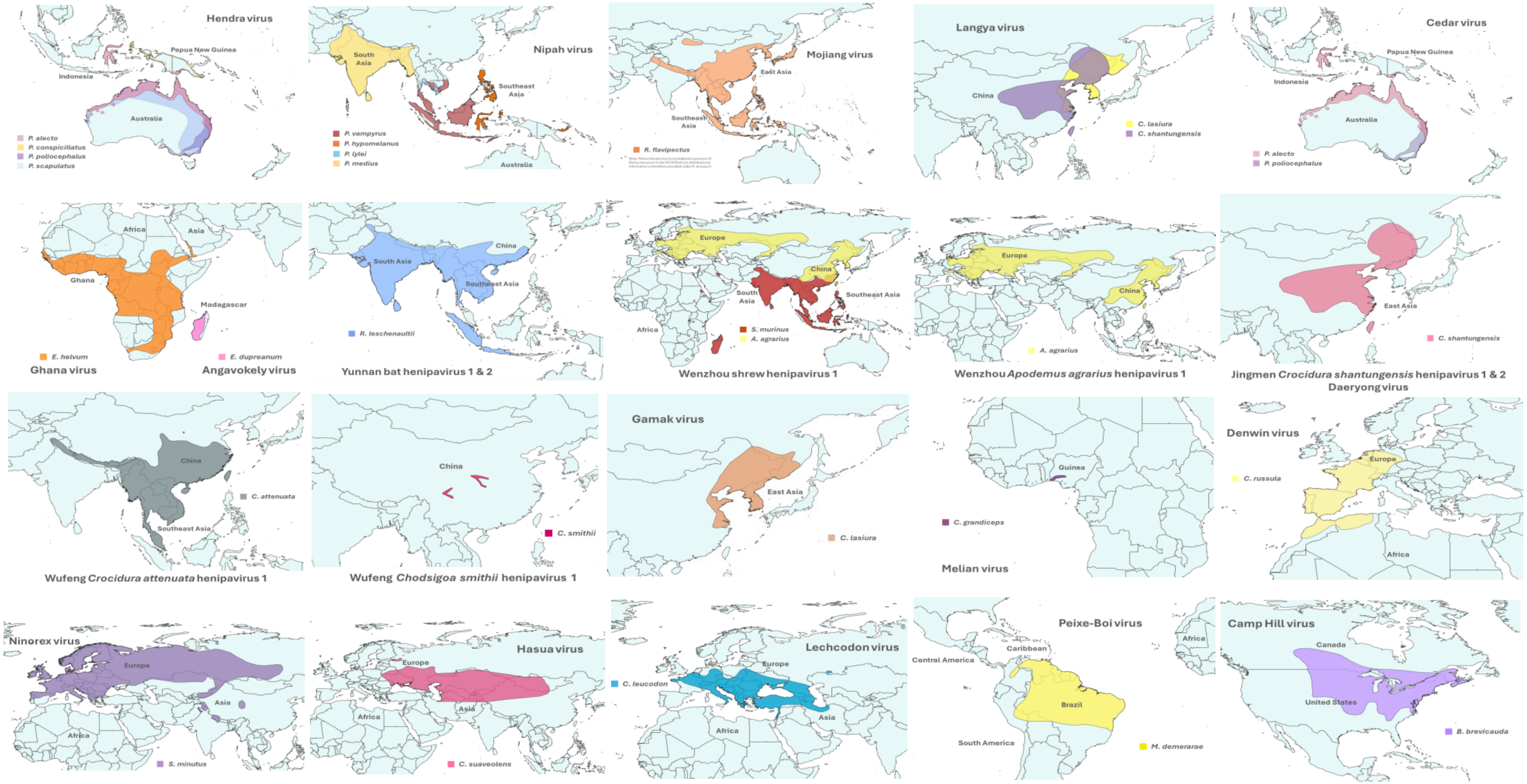

Supplementary figure 3. Henipaviruses and the geographical distribution of their animal hosts (listed in Table 1). Host range spatial data were derived from the International Union for Conservation of Nature (IUCN) Red List of Threatened Species (version 2025-1) (https://www.iucnredlist.org; accessed September 4, 2025). The mapped ranges represent the extent of occurrence of host species and are not indicative of population density or spillover risk, and these maps are not risk predictions.

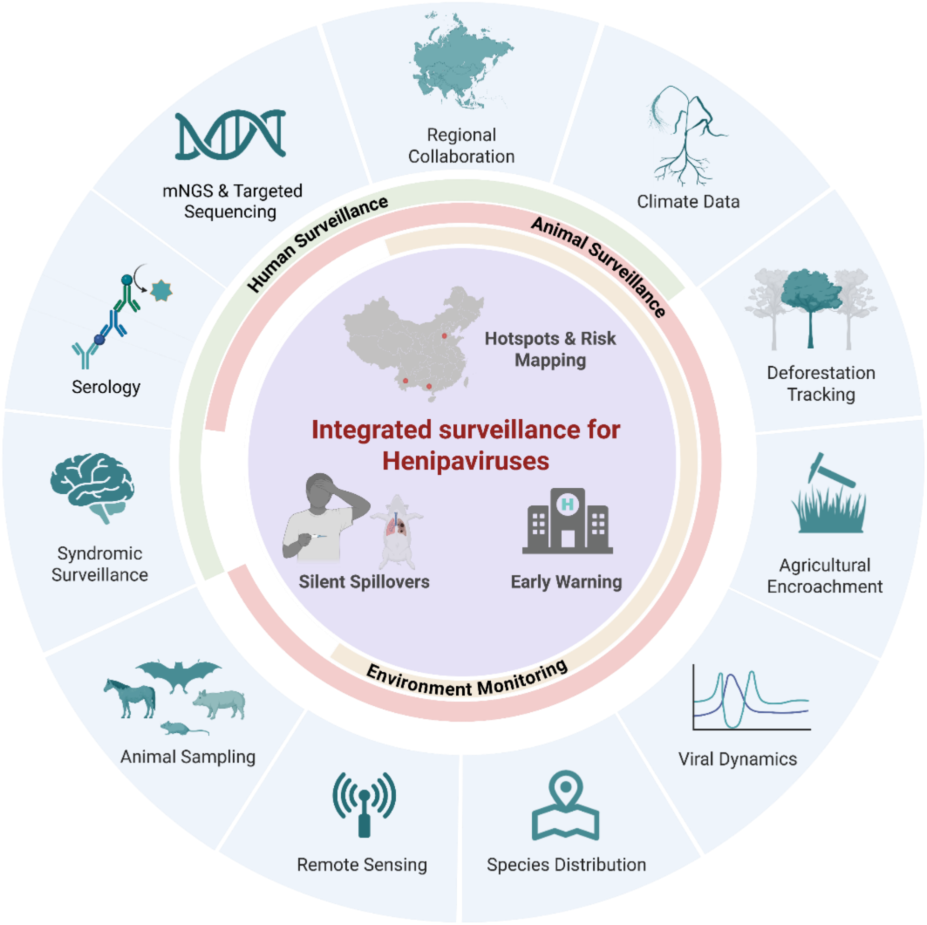

Supplementary figure 4. An integrated surveillance framework for Nipah virus, Hendra virus, and related henipa-like orthoparamyxoviruses, encompassing coordinated monitoring in humans, animals, and the environment. This approach aims to strengthen early detection of spillover events, facilitate identification of transmission hotspots, and support outbreak early warning systems for public health response and cross-border data governance.

Supplementary method 1. Spatial data sources, processing, threshold classification, and code availability.

Data sources

Global country and territory boundaries were obtained from the Natural Earth Admin-0 dataset (1:10 million scale) (version 5.1.1). Species distribution shapefiles for all confirmed natural hosts of henipaviruses were obtained from the International Union for Conservation of Nature (IUCN) Red List of Threatened Species (version 2025-1) spatial data. Host shapefiles were filtered to include only polygons coded as *extant* or *probably extant* (PRESENCE = 1 or 2) and to exclude polygons associated with *introduced* or *captive* populations (ORIGIN ≠ 3 or 4). Antarctica was excluded from the analysis. Territories were treated at the ADMIN level as defined in the Natural Earth dataset, thereby preserving overseas territories and dependencies as separate spatial units.

Spatial processing

All spatial layers were re-projected into an equal-area Mollweide coordinate reference system (ESRI:54009) to allow valid areal comparisons across latitudes. Total land area (km^2) was computed for each administrative unit. IUCN host range polygons were dissolved into a single union geometry representing the combined distribution of all known natural hosts. This host-union layer was then intersected with the Natural Earth boundaries to calculate, for each country/territory, the area of overlap with host ranges (km^2).

Threshold classification

The percentage of each country/territory's land area overlapping with the host-union layer was calculated as:

$$Percent\ overlap\ = \frac{Intersecting\ area\ (km^2)}{Total\ land\ area\ (km^2)} \times 100$$

A jurisdiction was classified as "included" if its percent overlap exceeded a predefined threshold. Sensitivity analyses were performed at three thresholds: ≥1%, ≥5% (primary threshold used in the main analysis), and ≥10% of land area. For each threshold, a list of included countries/territories was generated.

Code availability

All analysis code developed is openly available at: https://github.com/hivresearch-droid/Henipa_host. The repository contains the full reproducibility pipeline, including:

> henipa_country_overlap.py – Python script for calculating per-country overlap with host ranges and classifying inclusion thresholds (⩾1%, ⩾5%, ⩾10%).
> Dockerfile.henipa and run_overlap.sh – containerized workflow for reproducible, one-command execution.
> environment-geostack.yml – Conda environment specification for users preferring a local installation.
> henipa_country_overlap_README.md – step-by-step instructions for both Docker and Conda workflows.

The repository also includes example outputs (threshold-specific country lists, master overlap tables, processing logs) sufficient to validate the findings. Redistribution of raw IUCN Red List shapefiles is not permitted under IUCN licensing; users must obtain these data directly from IUCN (https://www.iucnredlist.org/resources/spatial-data).

Supplementary table 1. Countries and territories whose land area overlaps with the combined ranges of natural hosts of henipaviruses and related henipa-like orthoparamyxoviruses, evaluated at thresholds of ≥1%, ≥5%, and ≥10%. A "Yes" entry indicates that the country or territory meets or exceeds the specified threshold; "No" indicates that it does not.

Continent	Admin (Country/Territory)	Include at ≥1%	Include at ≥5%	Include at ≥10%
Africa	Algeria	Yes	Yes	Yes
	Angola	Yes	Yes	Yes
	Benin	Yes	Yes	Yes
	Bir Tawil	No	No	No
	Botswana	Yes	No	No
	Burkina Faso	Yes	Yes	Yes
	Burundi	Yes	Yes	Yes
	Cabo Verde	No	No	No
	Cameroon	Yes	Yes	Yes
	Central African Republic	Yes	Yes	Yes
	Chad	Yes	Yes	No
	Comoros	No	No	No
	Democratic Republic of the Congo	Yes	Yes	Yes
	Djibouti	Yes	No	No
	Egypt	No	No	No
	Equatorial Guinea	Yes	Yes	Yes
	Eritrea	No	No	No
	Ethiopia	Yes	Yes	Yes
	Gabon	Yes	Yes	Yes
	Gambia	Yes	Yes	Yes
	Ghana	Yes	Yes	Yes
	Guinea	Yes	Yes	Yes
	Guinea-Bissau	Yes	Yes	Yes
	Ivory Coast	Yes	Yes	Yes
	Kenya	Yes	Yes	Yes
	Lesotho	Yes	Yes	Yes
	Liberia	Yes	Yes	Yes
	Libya	No	No	No
	Madagascar	Yes	Yes	Yes
	Malawi	Yes	Yes	Yes
	Mali	Yes	Yes	Yes
	Mauritania	No	No	No
	Morocco	Yes	Yes	Yes
	Mozambique	Yes	Yes	Yes
	Namibia	No	No	No
	Niger	Yes	No	No
	Nigeria	Yes	Yes	Yes
	Republic of the Congo	Yes	Yes	Yes
	Rwanda	Yes	Yes	Yes
	Senegal	Yes	Yes	Yes
	Sierra Leone	Yes	Yes	Yes
	Somalia	No	No	No
	Somaliland	No	No	No
	South Africa	Yes	Yes	Yes
	South Sudan	Yes	Yes	Yes
	Sudan	Yes	Yes	Yes
	São Tomé and Principe	No	No	No
	Togo	Yes	Yes	Yes
	Tunisia	No	No	No
	Uganda	Yes	Yes	Yes
	United Republic of Tanzania	Yes	Yes	Yes
	Western Sahara	No	No	No
	Zambia	Yes	Yes	Yes
	Zimbabwe	Yes	Yes	Yes
	eSwatini	Yes	Yes	Yes
Asia	Afghanistan	Yes	Yes	Yes
	Akrotiri Sovereign Base Area	No	No	No

	Armenia	Yes	Yes	Yes
	Azerbaijan	Yes	Yes	Yes
	Bahrain	No	No	No
	Bangladesh	Yes	Yes	Yes
	Baykonur Cosmodrome	Yes	Yes	Yes
	Bhutan	Yes	Yes	Yes
	Brunei	Yes	Yes	Yes
	Cambodia	Yes	Yes	Yes
	China	Yes	Yes	Yes
	Cyprus	No	No	No
	Cyprus No Mans Area	No	No	No
	Dhekelia Sovereign Base Area	No	No	No
	East Timor	Yes	Yes	Yes
	Georgia	Yes	Yes	Yes
	Hong Kong S.A.R.	Yes	Yes	Yes
	India	Yes	Yes	Yes
	Indian Ocean Territories	No	No	No
	Indonesia	Yes	Yes	Yes
	Iran	Yes	Yes	Yes
	Iraq	No	No	No
	Israel	Yes	Yes	Yes
	Japan	Yes	Yes	Yes
	Jordan	No	No	No
	Kazakhstan	Yes	Yes	Yes
	Kuwait	No	No	No
	Kyrgyzstan	Yes	Yes	Yes
	Laos	Yes	Yes	Yes
	Lebanon	Yes	Yes	Yes
	Macao S.A.R	Yes	Yes	Yes
	Malaysia	Yes	Yes	Yes
	Mongolia	Yes	Yes	Yes
	Myanmar	Yes	Yes	Yes
	Nepal	Yes	Yes	Yes
	North Korea	Yes	Yes	Yes
	Northern Cyprus	No	No	No
	Oman	No	No	No
	Pakistan	Yes	Yes	Yes
	Palestine	Yes	Yes	Yes
	Philippines	Yes	Yes	Yes
	Qatar	No	No	No
	Saudi Arabia	Yes	No	No
	Scarborough Reef	No	No	No
	Siachen Glacier	Yes	Yes	Yes
	Singapore	Yes	Yes	Yes
	South Korea	Yes	Yes	Yes
	Spratly Islands	No	No	No
	Sri Lanka	Yes	Yes	Yes
	Syria	Yes	No	No
	Taiwan	Yes	Yes	Yes
	Tajikistan	Yes	Yes	Yes
	Thailand	Yes	Yes	Yes
	Turkey	Yes	Yes	Yes
	Turkmenistan	Yes	Yes	Yes
	United Arab Emirates	No	No	No
	Uzbekistan	Yes	Yes	Yes
	Vietnam	Yes	Yes	Yes
	Yemen	Yes	Yes	No
Europe	Aland	Yes	Yes	Yes
	Albania	Yes	Yes	Yes
	Andorra	Yes	Yes	Yes
	Austria	Yes	Yes	Yes
	Belarus	Yes	Yes	Yes
	Belgium	Yes	Yes	Yes
	Bosnia and Herzegovina	Yes	Yes	Yes
	Bulgaria	Yes	Yes	Yes

	Croatia	Yes	Yes	Yes
	Czechia	Yes	Yes	Yes
	Denmark	Yes	Yes	Yes
	Estonia	Yes	Yes	Yes
	Faroe Islands	No	No	No
	Finland	Yes	Yes	Yes
	France	Yes	Yes	Yes
	Germany	Yes	Yes	Yes
	Gibraltar	No	No	No
	Greece	Yes	Yes	Yes
	Guernsey	Yes	Yes	Yes
	Hungary	Yes	Yes	Yes
	Iceland	No	No	No
	Ireland	Yes	Yes	Yes
	Isle of Man	No	No	No
	Italy	Yes	Yes	Yes
	Jersey	Yes	Yes	Yes
	Kosovo	Yes	Yes	Yes
	Latvia	Yes	Yes	Yes
	Liechtenstein	Yes	Yes	Yes
	Lithuania	Yes	Yes	Yes
	Luxembourg	Yes	Yes	Yes
	Malta	No	No	No
	Moldova	Yes	Yes	Yes
	Monaco	Yes	Yes	Yes
	Montenegro	Yes	Yes	Yes
	Netherlands	Yes	Yes	Yes
	North Macedonia	Yes	Yes	Yes
	Norway	Yes	Yes	Yes
	Poland	Yes	Yes	Yes
	Portugal	Yes	Yes	Yes
	Republic of Serbia	Yes	Yes	Yes
	Romania	Yes	Yes	Yes
	Russia	Yes	Yes	Yes
	San Marino	Yes	Yes	Yes
	Slovakia	Yes	Yes	Yes
	Slovenia	Yes	Yes	Yes
	Spain	Yes	Yes	Yes
	Sweden	Yes	Yes	Yes
	Switzerland	Yes	Yes	Yes
	Ukraine	Yes	Yes	Yes
	United Kingdom	Yes	Yes	Yes
	Vatican	Yes	Yes	Yes
North America	Anguilla	No	No	No
	Antigua and Barbuda	No	No	No
	Aruba	No	No	No
	Bajo Nuevo Bank (Petrel Is.)	No	No	No
	Barbados	No	No	No
	Belize	No	No	No
	Bermuda	No	No	No
	British Virgin Islands	No	No	No
	Canada	Yes	Yes	Yes
	Cayman Islands	No	No	No
	Costa Rica	No	No	No
	Cuba	No	No	No
	Curaçao	No	No	No
	Dominica	No	No	No
	Dominican Republic	No	No	No
	El Salvador	No	No	No
	Greenland	No	No	No
	Grenada	No	No	No
	Guatemala	No	No	No
	Haiti	No	No	No
	Honduras	No	No	No
	Jamaica	No	No	No

	Mexico	No	No	No
	Montserrat	No	No	No
	Nicaragua	No	No	No
	Panama	No	No	No
	Puerto Rico	No	No	No
	Saint Barthelemy	No	No	No
	Saint Kitts and Nevis	No	No	No
	Saint Lucia	No	No	No
	Saint Martin	No	No	No
	Saint Pierre and Miquelon	No	No	No
	Saint Vincent and the Grenadines	No	No	No
	Serranilla Bank	No	No	No
	Sint Maarten	No	No	No
	The Bahamas	No	No	No
	Trinidad and Tobago	No	No	No
	Turks and Caicos Islands	No	No	No
	US Naval Base Guantanamo Bay	No	No	No
	United States Minor Outlying Islands	No	No	No
	United States Virgin Islands	No	No	No
	United States of America	Yes	Yes	Yes
South America	Argentina	No	No	No
	Bolivia	Yes	Yes	Yes
	Brazil	Yes	Yes	Yes
	Brazilian Island	No	No	No
	Chile	No	No	No
	Colombia	Yes	Yes	No
	Ecuador	No	No	No
	Falkland Islands	No	No	No
	Guyana	Yes	Yes	Yes
	Paraguay	No	No	No
	Peru	Yes	Yes	Yes
	Southern Patagonian Ice Field	No	No	No
	Suriname	Yes	Yes	Yes
	Uruguay	No	No	No
	Venezuela	Yes	Yes	Yes
Oceania	American Samoa	No	No	No
	Ashmore and Cartier Islands	No	No	No
	Australia	Yes	Yes	Yes
	Cook Islands	No	No	No
	Coral Sea Islands	No	No	No
	Federated States of Micronesia	No	No	No
	Fiji	No	No	No
	French Polynesia	No	No	No
	Guam	No	No	No
	Kiribati	No	No	No
	Marshall Islands	No	No	No
	Nauru	No	No	No
	New Caledonia	No	No	No
	New Zealand	No	No	No
	Niue	No	No	No
	Norfolk Island	No	No	No
	Northern Mariana Islands	No	No	No
	Palau	No	No	No
	Papua New Guinea	Yes	Yes	Yes
	Pitcairn Islands	No	No	No
	Samoa	No	No	No
	Solomon Islands	No	No	No
	Tonga	No	No	No
	Tuvalu	No	No	No
	Vanuatu	No	No	No
	Wallis and Futuna	No	No	No
Seven seas (open ocean)	British Indian Ocean Territory	No	No	No
	Clipperton Island	No	No	No
	French Southern and Antarctic Lands	No	No	No
	Heard Island and McDonald Islands	No	No	No

	Maldives	Yes	Yes	Yes
	Mauritius	No	No	No
	Saint Helena	No	No	No
	Seychelles	No	No	No
	South Georgia and the Islands	No	No	No
Total	257	**144**	**139**	**136**

Supplementary table 2. Comparative readiness of molecular platforms for henipavirus detection. Values are approximate and based on published prototypes, laboratory-developed tests, and official guidance.

Platform	Approximate limit of detection	Time-to-result	Instrumentation	Cost/test* (USD)	Cold chain / stability	Validation status	Appropriate use case
Multiplex RT-PCR	10 copies/µL[1]	2-3 h; WHO target product profile <6 h (optimal <2 h)[2]	Real-time PCR thermocycler; trained staff	15-25	Requires -20°C storage	Lab-developed assays; no WHO PQ[#]	Clinical diagnosis, reference labs[3,4]
RT-LAMP	~100 pg pseudovirus RNA (~10^2-10^3 copies)[5]	30-60 min[5]	Simple heater / dye readout	5-10	Lyophilized formats possible	Research / lab-developed only	Field screening; low-resource labs (research use)
RT-RPA / RT-RAA	10^3 copies/µL synthetic RNA[6]	20-40 min at 39°C[6]	Portable incubator + lateral flow strips	5-10	Lyophilized pellets available[6]	Research prototypes only	Point of care / outbreak response (research use)
CRISPR-based	10^3 copies/µL[7]; single-copy prototype[8]	≤60-120 min[7,8]	Portable fluorescence reader / lateral flow device	5-15	Lyophilization under development	Research prototypes only	Future rapid field diagnostics (research use)

[*] Costs exclude nucleic acid extraction

[#] WHO PQ, World Health Organization Prequalification of In Vitro Diagnostics (https://extranet.who.int/prequal/vitro-diagnostics/prequalified/in-vitro-diagnostics)

References:

1. He W, Ma T, Wang Y, et al. Development and evaluation of a quadruple real-time fluorescence-based quantitative reverse transcription polymerase chain reaction assay for detecting Langya, Mojiang, Nipah, and Cedar viruses. *Biosaf Health* 2024; **6**(2): 80-7.
2. World Health Organization. WHO R&D Blueprint: Priority Diagnostics for Nipah - Use Cases and Target Product Profiles. 2019. https://www.who.int/docs/default-source/blue-print/call-for-comments/who-nipah-dx-tpps-d.pdf. (accessed August 31, 2025)
3. European Centre for Disease Prevention and Control. Factsheet on Nipah Virus Disease. 2023. https://www.ecdc.europa.eu/en/infectious-disease-topics/nipah-virus-disease/factsheet-nipah-virus-disease. (accessed July 23, 2025)
4. US Centers for Disease Control and Prevention. Nipah virus: Facts for Clinicians. 2024. https://www.cdc.gov/nipah-virus/hcp/clinical-overview/index.html. (accessed August 28, 2025)
5. Ma L, Chen Z, Guan W, Chen Q, Liu D. Rapid and Specific Detection of All Known Nipah virus Strains' Sequences With Reverse Transcription-Loop-Mediated Isothermal Amplification. *Front Microbiol* 2019; **10**: 418.
6. Pollak NM, Olsson M, Marsh GA, Macdonald J, McMillan D. Evaluation of three rapid low-resource molecular tests for Nipah virus. *Front Microbiol* 2022; **13**: 1101914.
7. Miao J, Zuo L, He D, et al. Rapid detection of Nipah virus using the one-pot RPA-CRISPR/Cas13a assay. *Virus Res* 2023; **332**: 199130.
8. Jin K, Huang P, Li B, et al. A Single-Copy Sensitive and Field-Deployable One-Pot RT-RPA CRISPR/Cas12a Assay for the Specific Visual Detection of the Nipah Virus. *Transbound Emerg Dis* 2024; **2024**: 4118007.
